# A novel in vitro three-dimensional retinoblastoma model for evaluating chemotherapeutic drugs

**Published:** 2012-05-31

**Authors:** Moutushy Mitra, Chandana Mohanty, Anju Harilal, Uma K. Maheswari, Sanjeeb Kumar Sahoo, Subramanian Krishnakumar

**Affiliations:** 1 Department of Ocular Pathology, Vision Research Foundation, Sankara Nethralaya, Tamil Nadu, India; 2 Laboratory of Nanomedicine, Institute of Life Sciences, Chandrasekharpur, Bhubaneswar, India; 3 CeNTAB, SASTRA University, Tanjore, India

## Abstract

**Purpose:**

Novel strategies are being applied for creating better in vitro models that simulate in vivo conditions for testing the efficacy of anticancer drugs. In the present study we developed surface-engineered, large and porous, biodegradable, polymeric microparticles as a scaffold for three dimensional (3-D) growth of a Y79 retinoblastoma (RB) cell line. We evaluated the effect of three anticancer drugs in naïve and nanoparticle-loaded forms on a 3-D versus a two-dimensional (2-D) model. We also studied the influence of microparticles on extracellular matrix (ECM) synthesis and whole genome miRNA-gene expression profiling to identify 3D-responsive genes that are implicated in oncogenesis in RB cells.

**Methods:**

Poly(D,L)-lactide-co-glycolide (PLGA) microparticles were prepared by the solvent evaporation method. RB cell line Y79 was grown alone or with PLGA–gelatin microparticles. Antiproliferative activity, drug diffusion, and cellular uptake were studied by 3-(4,5-dimethylthiazol-2-yl)-2,5-diphenyltetrazolium bromide, a yellow tetrazole (MTT) assay, fluorescent microscope, and flow cytometry. Extra cellular matrix (ECM) synthesis was observed by collagenase assay and whole genome miRNA-microarray profiling by using an Agilent chip.

**Results:**

With optimized composition of microparticles and cell culture conditions, an eightfold increase from the seeding density was achieved in 5 days of culture. The antiproliferative effect of the drugs in the 3-D model was significantly lower than in the 2-D suspension, which was evident from the 4.5 to 21.8 fold differences in their IC_50_ values. Using doxorubicin, the flow cytometry data demonstrated a 4.4 fold lower drug accumulation in the cells grown in the 3-D model at 4 h. The collagen content of the cells grown in the 3-D model was 2.3 fold greater than that of the cells grown in the 2-D model, suggesting greater synthesis of the extracellular matrix in the 3-D model as the extracellular matrix acted as a barrier to drug diffusion. The microarray and miRNA analysis showed changes in several genes and miRNA expression in cells grown in the 3-D model, which could also influence the environment and drug effects.

**Conclusions:**

Our 3-D retinoblastoma model could be used in developing effective drugs based on a better understanding of the role of chemical, biologic, and physical parameters in the process of drug diffusion through the tumor mass, drug retention, and therapeutic outcome.

## Introduction

A suitable three dimensional (3-D) culture system provides a more physiologically relevant approach to the analysis of gene function, regulation, and cell phenotype ex vivo [1]. It has previously been shown that engineering the cell culture micro-environment to create growth conditions that more accurately mimic the in vivo behavior of cells is an essential step for improving the predictive accuracy of the drug discovery process [2]. Evidence suggests that modification of cell growth conditions can radically influence the behavior of cells in response to chemical reagents [3]. Many important studies have been done on cell proliferation, differentiation, and function in vitro; however, experience with cells in a flat tissue culture flask is different from the complexities of tissues within the body. In tissues, cells connect to each other as well as to the extracellular matrix (ECM), which is a key regulator of normal homeostasis and tissue phenotype [1]. Receptor complexes on the surface of cells facilitate interactions with their neighbors, with the ECM, and with other exogenous factors to enable cells to interpret the multitude of biochemical and physical cues from the immediate environment. The metastatic potential of tumor cells is believed to be regulated by interactions between the tumor cells and their extracellular environment (i.e., ECM). These interactions can be modified by the accumulation of genetic changes and by the transient alterations in gene expression induced by the local tumor microenvironment [4]. Given this intricate mechanical and biochemical interplay; important biologic properties may be missed if cells are studied within an in vitro culture system [5]. It has also been suggested that many important signals, key regulators, and tissue phenotypes are lost when cells are cultured on substrates such as culture plates [6]. Therefore, developing an in vitro model of a tumor that in many aspects would resemble an actual tumor to obtain a realistic assessment of drug efficacy before their testing in animal models or patients is important [7]. Based on previous reports [7] we hypothesized that the cells grown with poly(D,L)-lactide-co-glycolide (PLGA)–gelatin microparticles behave differently than cells grown without PLGA–gelatin microparticles because of the structural, molecular, and genetic variations in cells grown in two different structural configurations. We selected three model anticancer drugs (doxorubicin, carboplatin, and etoposide), which are currently used in clinical practice for the treatment of retinoblastoma, and drug-loaded nanoparticles to determine the drug efficacy in both models. We studied drug diffusion and cellular uptake to understand the discrepancy in drug efficacy in cells grown with or without PLGA–gelatin microparticles. In addition we studied the influence of microparticles on ECM synthesis and whole genome miRNA-microarray profiling to identify 3-D-responsive genes that have been implicated in oncogenesis, survival, and growth of in vivo tumor.

## Methods

### Materials

PLGA (copolymer ratio 50:50, molecular weight [MW]=34,000 Da, inherent viscosity=0.41) was purchased from Birmingham polymers, Inc. (Birmingham, AL). Carboplatin, doxorubicin hydrochloride (DOXHCL), etoposide, polyvinyl alcohol (PVA; average MW 30,000–70,000 Da), BSA (fraction V), sucrose, chitosan from crab shells (85% deacetylated), and gelatin were purchased from Sigma-Aldrich (St. Louis, MO). All other chemicals used were of analytical grade obtained from Sigma-Aldrich. Media and fetal bovine serum (FBS) were purchased from Gibco-BRL (Rockville, MD). Y79 cells were cultured in RPMI 1640 medium (Biocolor Ltd, County Antrim, UK). The Sircol Soluble Collagen Assay kit was purchased from Biocolor Ltd. TRIZOL reagent was purchased from Invitrogen (Carlsbad, CA). TURBO DNase was purchased from Ambion (Genetix Biotech Asia Pvt. Ltd., New Delhi, India).

### Preparation of different drug-loaded nanoparticles

#### Carboplatin*-*loaded nanoparticles

Nanoparticles containing carboplatin (CNPs) were prepared as decribed by Parveen et al. [8]. Briefly, an aqueous solution of 2 ml sodium alginate (0.625 mg/ml) was added dropwise into 8 ml of chitosan solution (0.5 mg/ml dilute solution of HCl, with pH adjusted to approximately 5.5 with 0.1 M NaOH) containing carboplatin (1.25 mg). After overnight stirring of the emulsion, the CNPs were recovered by ultracentrifugation (Sorvall Ultraspeed Centrifuge; Kendro, Asheville, NC) at 30,000× g for 30 min at 4 °C. The nanoparticles were formed as a result of the interaction between negative groups of alginate and positively charged amino groups of chitosan. The pellets were dispersed in double-distilled water and lyophilized (LYPHLOCK 12; Labconco, Kansas City, MO) for 48 h and then stored at 4 °C for further studies.

#### Etoposide-loaded nanoparticles

Etoposide-loaded nanoparticles (ENP) were formulated by using the single emulsion–solvent evaporation technique. In this method, etoposide (equivalent to 10% weight [w]/w dry weight of polymer) was dissolved in 3 ml organic solvent (chloroform) containing 90 mg of polymer (PLGA) to form a primary emulsion. The emulsion was further emulsified in an aqueous PVA solution (12 ml, 5% w/volume [v]) to form an oil-in-water emulsion. The emulsification was performed using a microtip probe sonicator (VC 505; Vibracell Sonics, Newton, CT) at 39 W of energy for 2 min in an ice bath. The emulsion was stirred using a magnetic stirrer overnight to evaporate the organic solvent. The resultant nanoparticles were ultracentrifuged at 125,000× g for 20 min at 4 °C with twice washing with double-distilled water to remove the excess amount of PVA and unencapsulated etoposide. The obtained particles were resuspended in double-distilled water and lyophilized for 48 h to obtain the powder form and then stored at 4 °C for further use.

#### Doxorubicin-loaded nanoparticles

Doxorubicine-loaded nanoparticles (DNP) were prepared by using the single emulsion–solvent evaporation technique. In brief, a solution of 100 mg of PLGA polymer and 10 mg of doxorubicin (DOX) in 3 ml of 12.5% (v/v) methanol in chloroform solution was emulsified in 12 ml of 2% w/v aqueous solution of PVA to form an oil-in-water emulsion. The emulsification was performed using a microtip probe sonicator set at 55 W of energy output for 2 min over an ice bath. The emulsion was stirred overnight at room temperature on a magnetic stirring plate to allow evaporation of the organic solvent and formation of DNPs. DNPs were recovered by ultracentrifugation at 125,000× g for 20 min at 4 °C, washed twice with double-distilled water to remove unbound PVA and unencapsulated drug, and then lyophilized for 2 days to obtain the powdered DNPs. The lyophilized DNPs were stored at 4 °C until further use.

### Formulation of gelatin microparticles

PLGA microparticles were prepared by the solvent evaporation method with slight modifications [9]. In brief, 800 μl of an aqueous phase (W1) was prepared with BSA (2.5% w/v), sucrose (10% w/v), chitosan (1.25% w/v), gelatin (5% w/ v), and PVA (5% w/v) and emulsified with polymer solution (O; 200 mg PLGA in 4 ml of dichloromethane), using an homogenizer (Biospec Product Inc., Bartlesville, OK) at 80× g to form an oil-in-water primary emulsion. The resultant primary emulsion was added dropwise into a 1% w/v aqueous solution of PVA containing 10% w/v sucrose (W2) under constant magnetic stirring on a stirring plate to form a multiple emulsion (W1/O/W2). The emulsion was stirred overnight on a magnetic stir plate to evaporate the organic solvent. The resultant microparticles were recovered by centrifugation at 15,000× g, washed three times with distilled water, and then lyophilized for 48 h the lyophilized microparticles were stored at 4 °C until further use.

### Characterization of different drug-loaded nanoparticles

Mean particle size and size distribution of the different drug-loaded nanoparticles were determined by using a Malvern Zeta-sizer Nano ZS (Malvern Instrument, Malvern, Worcestershire, UK) based on quasi-elastic light scattering. Briefly, approximately 1 mg/ml of different formulations of the nanoparticle solutions were prepared in double-distilled water and sonicated for 30 s in an ice bath. Size measurements were performed in triplicate following the dilution (100 ml diluted to 1 ml) of the NP suspensions in MilliQ water at 25 °C. The Zeta potential was measured in the same instrument at 25 °C using the above protocol. All measurements were performed in triplicate.

### Characterization of gelatin microparticles

The shape and surface morphology of gelatin microparticles were characterized by scanning electron microscopy (SEM, Hitachi S-3400N; Hitachi High-Technologies, Krefeld, Germany). The powdered microparticles were attached to a brass stub through double adhesive tape and were gold coated using a sputter gold coater at 20 KV (Hitachi, E-1010, Ion Sputter; Hitachi High-Technologies). The stub was fixed in a sample holder and placed in the vacuum chamber of the SEM (Hitachi S-3400N; Hitachi High-Technologies) and observed under low vacuum. The average particle diameters of microparticles were determined from the SEM picture.

### Cell culture and cell seeding for gelatin microparticle composition optimization

Y79 cells RCB1645 were obtained from the RIKEN cell bank (Ibaraki, Japan) and maintained in RPMI-1640 media supplemented with 10% FBS and 1% penicillin streptomycin in T-75 cm^2^ flasks in an incubator (Thermo Electron Corporation, Asheville, NC) at 37 °C and 5% CO_2_. Fresh retinoblastoma tumor cells were isolated from RB-enucleated eyeballs (Vision Research Foundation, Sankara Nethralaya, Chennai, India) and cultured as described above. All samples were collected with the approval of the institutional review board at VRF (Vision Research Foundation) and in accordance with the Declaration of Helsinki. Each formulation of PLGA microparticles was weighed (approximately 2 mg/ml) and soaked for 3 h at 37 °C in 70% alcohol. Microparticles were centrifuged to remove alcohol and washed twice with media. Fifty percent FBS was added, and the solution was kept in a CO_2_ incubator for 3 h.

For studying the effects of microparticle composition on cell growth, 1 ml of the microparticle suspension prepared as above was transferred to separate wells of 6-well plates. The medium from each well was aspirated carefully, leaving microparticles in the wells before cell seeding. A 500-μl aliquot of Y79 cell suspension (1×10^6^ cells/ml)/fresh RB cell suspension in RPMI 1640 medium was added directly onto microparticles in each well. After 3 h of incubation at 37 °C in a CO_2_ incubator, an additional 1.5 ml of medium was added to each well and cells were allowed to grow in the incubator. The medium was changed on alternate days, and cells were counted at 7 days post seeding to determine the effect of microparticle composition on Y79 cell growth. To study the total number of cells grown in gelatin microparticles, the adherence cells from a microparticle clump was recovered after 10 days of post seeding. Cell detachment was done by treating the microparticles with 1 ml of 0.1 M citrate buffer containing 0.1% crystal violet for 1 h at 37 °C. After detachment, the total number of cells grown with a different formulation of microparticles was counted separately using a hemocytometer. Photomicrographs of selected plates with cells attached to microparticles were taken with a phase-contrast inverted microscope fitted with a digital camera. The fluorescent images of Y79/RB cells labeled with CellTracker™ CM-DiI reagent (Molecular probes, Invitrogen, Bangalore, India) grown with the scaffold were captured.

### Antiproliferative effects of anticancer drugs and drug-loaded nanoparticles

The antiproliferative effects of drugs, both in the native form and encapsulated inside nanoparticles, were analyzed using the methodology of Horning et al. [7]. For this study the Y79/RB cells were allowed to grow in a 2-D suspension (tissue culture-treated Petri dishes) and a 3-D model (in microparticles). The tissue culture-treated Petri dishes (100 mm×20 mm; #353003; Becton Dickson, Franklin Lakes, NJ) taken for the 2-D study provided more surface area for cells to grow without reaching confluency. For the 3-D model, nontissue culture Petri dishes (100 mm×15 mm; #08–757–13; Fisher Scientific, Mumbai, India) were used; these enabled better cellular attachment and growth onto microparticles compared to the surface of a Petri dish. Cell seeding in the case of the 2-D monolayer was performed the second day post seeding on microparticles at a cell density of 0.5×10^6^ cells/ml. This protocol for cell seeding and growth was optimized to obtain approximately the same cell count in both sets of experiments at the time of drug treatment for a better comparison of drug efficacy. Three model anticancer drugs, i.e., doxorubicin, carboplatin and etoposide, were selected to determine their IC_50_ values in Y79/RB cells grown on 2-D as well as 3-D models. The chosen drugs are currently being used in the treatment of retinoblastoma and are well known DNA intercalating anticancer agent. For the cell toxicity study the stock solutions of drugs were prepared in DMSO and diluted appropriately in tissue culture medium to obtain the desired concentration [8]. Similarly, equivalent concentrations of drug encapsulated in nanoparticles (doxorubicin and etoposide in PLGA nanoparticles, carboplatin in chitosan nanoparticles) were prepared with RPMI medium. On day 5 of post seeding, media was carefully removed and replaced with 5 ml of media containing different concentrations of drugs and drug-loaded nanoparticles. After 48 h the treated cells (2-D and 3-D culture) were collected and filtered through 35 µm nylon mesh to separate cells from microparticles. The detached cells were stained using 0.4% (w/v) trypan blue in deionized water, and viable cells (unstained) were counted using a hemocytometer.

### Collagen assay

Collagen content was assayed using the manufacturer’s protocol. Cells with/without PLGA–gelatin microparticles were suspended in PBS (137 mM NaCl, 2.7 mM KCl, 10 mM sodium phosphate dibasic, 2 mM potassium phosphate monobasic and a pH of 7.4). The preparations were centrifuged at 80× g using an Eppendorf microcentrifuge (5417R; Eppendorf-Netheler-Hinz-GmbH, Hamburg, Germany), and the sediment was treated with 1 ml of 0.5 M acetic acid for 18 h at 4 °C. One milliliter of the dye reagent supplied with the assay kit was added to 100 μl of the acid extract in 1.5 ml Eppendorf tubes and mixed gently for 30 min at room temperature. The collagen-bound dye complex was recovered after centrifugation at 7,800 ×g for 10 min as above. The complex was solubilized in 1 ml of the alkali reagent provided with the assay kit. The absorbance of the samples was measured at 550 nm using a microwell plate reader (Fisher Biotech, Pittsburgh, PA). A standard plot of collagen was prepared under similar conditions. Either microparticles without cells or PBS was used as controls. The amount of collagen present in the native microparticles was deduced from the actual collagen amounts obtained in 2-D and 3-D culture experiments. Therefore the there was no interference of the polymer used in microparticles in the assay.

### Recovery of cells from poly(D,L)-lactide-co-glycolide–gelatin microparticles following freezing

To determine whether the cells grown in the 3-D model could be rescued after freezing, microparticles were seeded (seeding density) 0.5×10^6^ cells/mg of microparticles) and cultured as above in noncell culture Petri dishes for 5 days. Cells with PLGA–gelatin microparticles were washed with RPMI-1640 medium, resuspended in 1 ml of cryopreservation media (DMSO, 10%; FBS, 90%), transferred into cryovials, and then frozen in liquid nitrogen. After 24 h of freezing, cells were thawed and the entire content of each cryovial was transferred into Petri dishes and recultured for 48 h in RPMI medium. Cells with PLGA–gelatin microparticles cultured as above for the same time period but not subjected to the freezing step, were used as the control. Cells cultured without PLGA–gelatin microparticles (0.5×10^6^ cells/ml of cryopreservation media) were also frozen and recultured as above. Cell viability for each sample was determined using trypan blue as above. The percentages of cells with or without PLGA–gelatin microparticles rescued were calculated from the cell numbers with and without the freezing step.

### Flow cytometry

Doxorubicin was used for this study because of its inherent fluorescent property. Fluorescent intensity as a measure of drug uptake by cells with or without PLGA–gelatin microparticles was determined using a FACSCalibur flow cytometer (Becton Dickson) at 488 nm excitation and a 585/42 filter (564–606 nm) to match the emission spectra of doxorubicin. On day 5 post seeding, media were carefully removed and 5 ml of doxorubicin solution (2,500 ng/ml) was added to each Petri dish and placed at 37 °C for 4 and 8 h each with triplicates. Cells were trypsinized from microparticles and monolayered as described above, centrifuged, and resuspended into a single cell suspension in DPBS + 2% FBS at concentrations of either 2×10^6^ cells/ml or 1×10^6^ cells/ml. The cell suspensions containing microparticles were filtered twice through a 35-μm nylon mesh tube cap into a 12×75 mm round-bottom tube (#352235; Becton Dickson) to remove microparticles from the cell suspension.

### Oligonucleotide arrays

Total RNA used for the microarray analysis was isolated from cultured cells using TRIZOL reagent and treated with TURBO DNase to remove the DNA contamination. The RNA samples (10 μg each) in a 50-μl reaction were treated with 1 μl of TURBO DNase (2 U) in 1× TURBO DNase buffer at 37 °C for 30 min. After incubation, the RNA sample was extracted with phenol–chloroform to inactivate the TURBO DNase. Agilent's Low RNA Input Linear Amplification Kit PLUS (Agilent Technologies Genotypic, Bangalore, India) was used to generate fluorescent complementary RNA (cRNA). This method uses T7 RNA polymerase, which simultaneously amplifies the target material and incorporates cyanine 3-labeled cytidine tri-phosphate. Qiagen’s RNeasy mini spin columns (Qiagen, New Delhi, India) were used for purification of the amplified cRNA samples, and the samples were then hybridized to the Human Whole Genome 44K Oligo Microarray for 17 h at 65 °C, as recommended by the manufacturer (Agilent Technologies). Data analysis was done using Genespring GX version 10 (Agilent Technologies). Agilent Feature Extraction software (G25677AA; Agilent Technologies) was used to analyze the microarray data. Two biologic replicates were used for gene expression microarray analysis.

### MicroRNA isolation and expression analysis

#### RNA extraction

Total RNA was isolated using TRI-Reagent (Ambion, Austin, TX). RNA concentration and purity were quantified using a Nanodrop (Nanodrop, Santa Clara, CA) spectrophotometer with the nanodrop ribogreen assay, and the quality of the total RNA was determined on an Agilent bioanalyzer (Agilent, Santa Clara, CA).

#### MicroRNA array hybridization and detection

Two biologic replicate samples were used for the miRNA microarray analysis. Human miRNA V2 8×15k Agilent arrays, which represent 723 human and 76 human viral miRNAs, were used for the miRNA analysis. Total RNA underwent phosphatase treatment. The 3′ end of the dephosphorylated RNA was ligated with one molecule of cyanine 3-cytidine bisphosphate (3-pCp), as this reagent selectively labels and hybridizes mature miRNAs with greater than 30% efficiency. A hybridization cocktail was added to the arrays, and hybridization was performed in the hybridization oven set at 55 °C for 20 h. The microarrays were washed using Agilent wash buffer. Scanning and extraction was performed using Agilent feature extraction software. The study was done in three biologic replicates. Data analysis was done using Genespring GX version 10. Agilent Feature Extraction software (G25677AA) was used to analyze the microarray data. The sum of background-subtracted signals for each repeated miRNA was calculated and log transformed to log base 2. The cut off was greater than 1 (log-transformed value) and less than 1 (log-transformed value).

#### Real-time quantitative reverse-transcriptase (RT)-PCR

RNA was extracted by the guanidine isothiocyanate and chloroform method (TRI Reagent; Sigma-Aldrich, Bangalore, India). All RNA samples were treated with DNase (Turbo; Ambion, Genetix Biotech Asia Pvt. Ltd). For all samples, 1 μg of total RNA was used to synthesize first-strand cDNA with reverse transcriptase (SuperScript II; Invitrogen, Joyvel, Chennai, India) and random primers. The cDNA synthesis was performed at 37 °C for 60 min after heat inactivation at 95 °C for 10 min. The primer sequences of the selected genes from the microarray are listed in Table 1. PCR was performed using 1× SYBR Green PCR Master Mix (Applied Biosystems, Lab India, Chennai, India) on a real-time PCR system (Prism 7300; Applied Biosystems). Cycling conditions were as follows: 2 min at 50 °C, 10 min at 95 °C, and 40 cycles of 15 s at 95 °C, plus 1 min at 60 °C. Commercial software (SDS ver.2.3; Applied Biosystems) was used to calculate ΔΔCt relative expression values for all the genes studied, normalized to the GAPDH endogenous control.

**Table 1:** List of Primers used for Real time Quantitative RT–PCR.

**Gene name**	**Primer sequences**	**Tm**
*ABCC6*	FP 5′TTGGATTCGCCCTCATAGTC 3′	65 °C
	RP 3′GGTAGCTGGCAAGACAAAGC 5′	
*HMGA2*	FP 5′GGCCAGCTCATAAAATGGAA 3′	61 °C
	RP 3′TACTGTTCCATTGGCCACAA 5′	
*MMP9*	FP 5′TTGACAGCGACAAGAAGTGG 3′	64 °C
	RP 3′GCCATTCACGTCGTCCTTAT 5′	
*ABCA3*	FP 5′AGGAAAGGAGGCTGAAGGAG 3′	65 °C
	RP 3′GTGCTGACCATGAAGCTGAA 5′	
*ERBB3*	FP 5′GCCAATGAGTTCACCAGGAT 3′	64 °C
	RP 3′ACGTGGCCGATTAAGTGTTC 5′	
*BCL2*	FP 5′GGATGCCTTTGTGGAACTGT 3′	63 °C
	RP 3′AGCCTGCAGCTTTGTTTCAT 5′	
*MYCN*	FP 5′CTTCGGTCCAGCTTTCTCAC 3′	64 °C
	RP 3′GTCCGAGCGTGTTCAATTTT 5′	
*APAF-1*	FP 5′TTCTGATGCTTCGCAAACAC 3′	63 °C
	RP 3′CTGGCAAATCTGCCTTCTTC 5′	
*CDKN2A*	FP 5′ATATGCCTTCCCCCACTACC 3′	67 °C
	RP 3′CCCCTGAGCTTCCCTAGTTC 5′	
*CDKN1A*	FP 5′ATGAAATTCACCCCCTTTCC3′	65 °C
	RP 3′CCCTAGGCTGTGCTCACTTC5′	
*PDCD2*	FP 5′GCATTGCCACCATAAATCCT3′	63 °C
	RP 3′GCAGTTTCCCATATGGTGCT5′	

### Statistical analysis

The statistical analysis included the independent Student *t* test. Statistical analysis was performed using SPSS version 12.0 software (Chicago, IL). P values less than 0.05 were considered significant.

## Results

### Characterization of different drug-loaded nanoparticles

**Figure 1:**
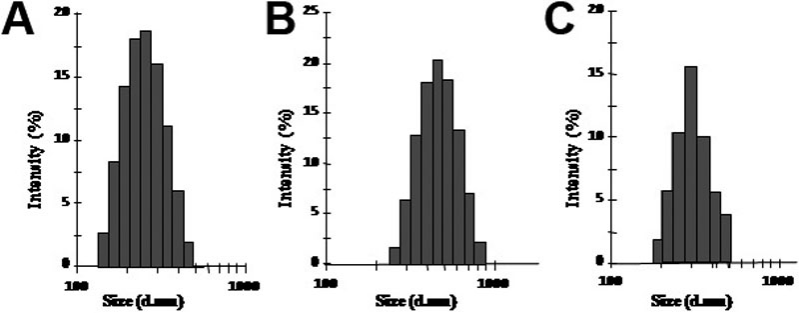
This figure shows the size distribution of different drug-loaded nanoparticles measured by dynamic light scattering analysis. **A**: Etoposide loaded nanoparticles (ENPs) showing an average diameter of 256 nm. **B**: Carboplatin loaded nanoparticles (CNPs) showing an average diameter of 507 nm. **C**: Doxorubicin loaded nanoparticles (DNPs) showing an average diameter of 249 nm.

The size distribution and surface charge of different drug-loaded nanoparticles were measured by dynamic light scattering analysis. This study revealed that ENPs, CNPs, and DNPs have an average diameter of 256 nm, 507 nm, and 249 nm, respectively, as shown in Figure 1 and have an average Zeta potential of −13 mV, –36 mV, and −5.56 mV, respectively.

### Formulation and characterization of poly(D,L)-lactide-co-glycolide microparticles

**Figure 2:**
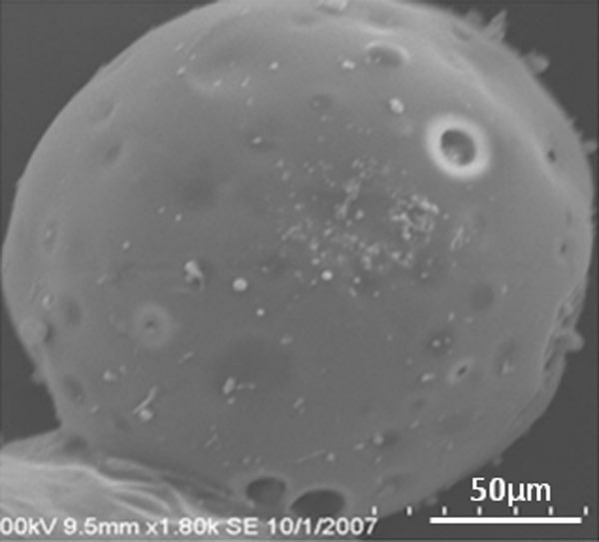
This figure shows the scanning electron microscope picture of a Poly(D,L-lactide-co-glycolide) (PLGA) scaffold microparticle containing 1.25% chitosan and 5% gelatin (100 mg PLGA, 1.25 mg chitosan, and 5 mg gelatin). Surface morphology of microparticles was characterized by scanning electron microscopy (SEM). The average diameter of formulated microparticle in this study ranged from 145 μm to 162 μm.

In the current study, we prepared a 3-D tumor model with PLGA–gelatin microparticles for in vitro evaluation of different anticancer drugs (native and loaded in nanoparticles). It was previously well documented that gelatin is a suitable candidate for scaffold materials because of its biocompatibility, low immunogenicity, and biodegradability. Gelatin contains Arg–Gly–Asp (RGD)-like sequences that promote cell adhesion and migration by forming a polyelectrolyte complex. Furthermore, the anionic cell surface provided by the gelatin–chitosan-blended microparticles provide better cell adhesion and cellular bioactivity for proliferating cells. A successful microparticle formulation was achieved with a highly porous matrix to facilitate the infiltration of proliferating cells. As evidenced from the SEM image, the formulated microparticle showed a porous infrastructure with interconnected void structures and had a spherical shape covered with a thin film of polymer (Figure 2). This rough surface could be due to deposition of cationic chitosan on the anionic microparticle surface, which is apparent from the surface characteristics [7]. Similarly, particle size is an important parameter that could affect the degradation of the polymer matrix [10]. To achieve a microparticle of a desired diameter, the conditions for emulsification and formulation composition were optimized. The average diameter of the formulated microparticle in this study ranged from 145 μm to 162 μm.

### Effect of composition of the scaffold and influence of cell seeding density on Y79 cell growth

**Figure 3:**
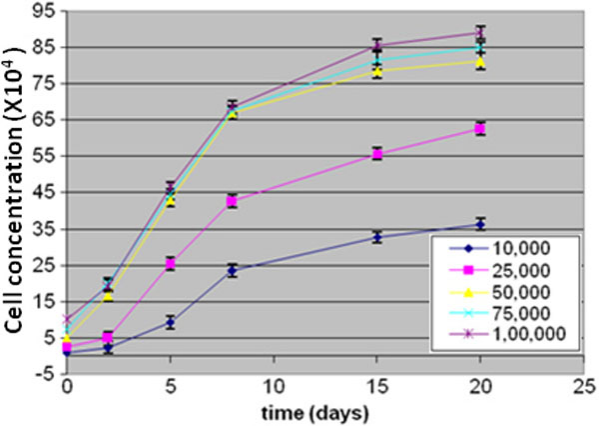
This figure shows the growth kinetics of Y79 cells with different seeding densities (10^4^ to 10^5^ cells) co-cultured with a gelatin scaffold for 20 days. The cells were harvested and counted on 2nd, 5th, 8th, 15th, and 20th day. The Y79 growth kinetics results on gelatin microparticle demonstrated the composition of formulated microparticle facilitated profound Y79 cell proliferation.

**Figure 4:**
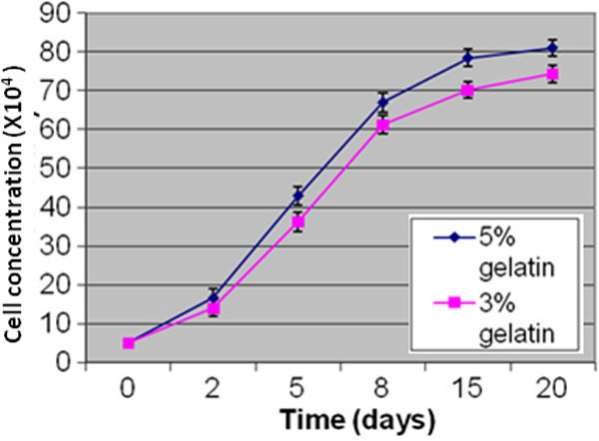
This figure shows the growth kinetics of Y79 cells co-cultured with microparticles formulated with different concentrations of gelatin (3% and 5% gelatin). The graph shows increased cell concentrations on 15th and 20th. Day of Y79 cell culture on microparticles coated with 5% gelatin (p<0.01) when compared to that of 3% gelatin coated microparticles 0.01).

We studied the effect of gelatin concentration (used in microparticle composition) on the proliferation of Y79 cells. The Y79 growth kinetic results on the gelatin microparticle demonstrated that the composition of the formulated microparticle facilitated profound Y79 cell proliferation (Figure 3). It was further seen that the microparticles formulated with a higher gelatin content (5% w/v) demonstrated better cell growth than those formulated with a lower gelatin content (3% w/v; Figure 4). Therefore, PLGA microparticles fabricated with 5% gelatin were chosen as a suitable formulation for further studies.

**Figure 5:**
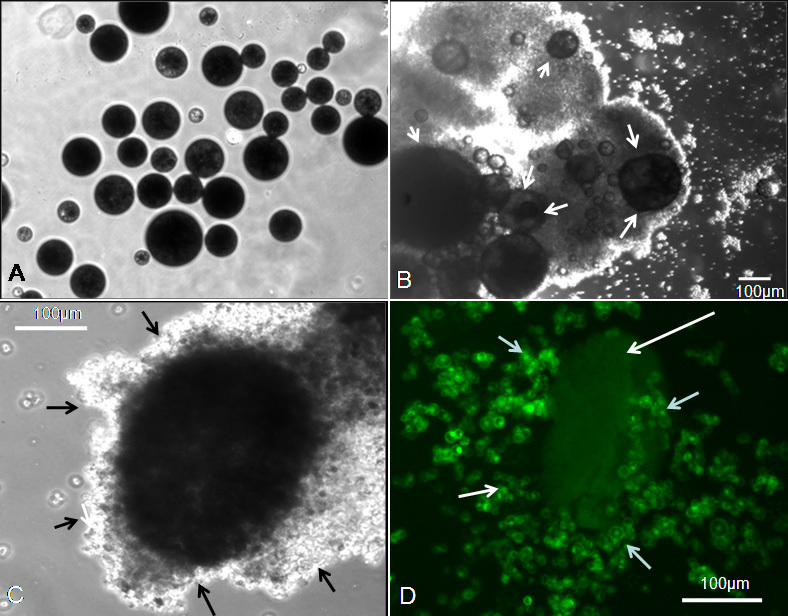
This figure shows the phase contrast and fluorescent microscope images of Y79 cells grown on Poly(D,L-lactide-co-glycolide) (PLGA) scaffold microparticles. **A**: Phase contrast microscope picture of Gelatin-coated PLGA microparticles under 10× magnification. **B**: Phase-contrast images showing cells attached to the microparticles (white arrows pointing Y79 cells attached to microparticles) forming a three-dimensional growth over the microparticles under 10× magnification. **C**: Phase contrast microscopic image of 3-D growth of Y79 cells over scaffold microparticles (black arrow pointing to Y79 cells attaché to microparticles) under 40× magnification. **D**: Fluorescent microscopic image showing the 3-D growth of Y79 cells (labeled with Celltracker dye) over scaffold microparticle (white arrows pointing Y79 cells attached to microparticle) under 40× magnification.

The optimized composition of microparticles, which consisted of 5% gelatin and 1.25% chitosan, demonstrated cell growth from an initial seeding cell density of 0.05×10^6^ to a cell density of 0.4×10^6^ cells/mg of microparticles, which is an eightfold increase in cell density after 6 days of culture. Cells attached more to the microparticles rather than to the surface of the nontissue culture Petri dish. Initially, cells were seen attached to the microparticle surface, and with time cells engulfed the microparticles completely, forming a 3-D tumor-like structure (Figure 5A-C phase contrast and Figure 5D fluorescence-labeled Y79 cells). Either a single microparticle or a cluster of two to three microparticles were witnessed to form the 3-D tumor-like structure.

### Extracellular matrix synthesis and retrieval of cells from a three-dimensional model following freezing

**Figure 6:**
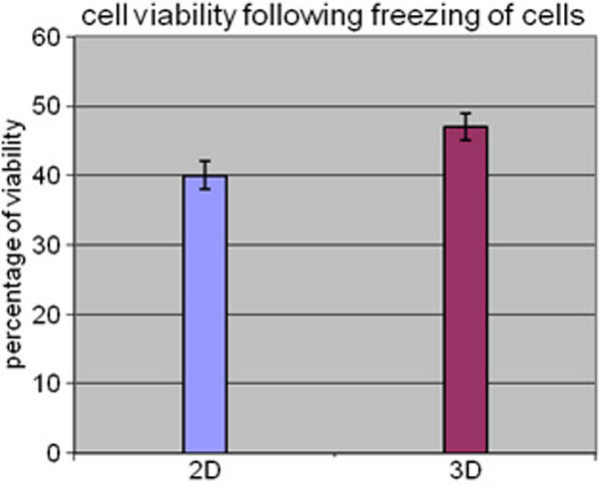
This figure shows the comparison of post freeze and thaw viability of Y79 cells between Y79 cell culture without microparticles (2-D) and Y79 cells co-cultured with microparticles (3-D). The bar diagram shows the viability of Y79 cultured cells with (3-D) and without (2-D) microparticles retrieved from freezing. The Y79 cells viability was slightly higher in 3-D model compared to that in 2-D monolayer (41% versus 35%). Error bars represent standard deviation obtained from triplicates.

After 5 days of growth, cells in the 3-D model synthesized 59.8±1.2 µg of collagen per 1×10^6^ cells, which was significantly higher than the 25.29±0.7 µg of collagen synthesized for the same number of cells in the 2-D monolayer (p<0.05, n=5). Following the freezing step, cell viability was slightly higher in the 3-D model compared to that in the 2-D monolayer (41% versus 35%; Figure 6).

### Antiproliferative activity of drugs and drug-loaded nanoparticles on Y79 cells grown with or without a scaffold

**Figure 7:**
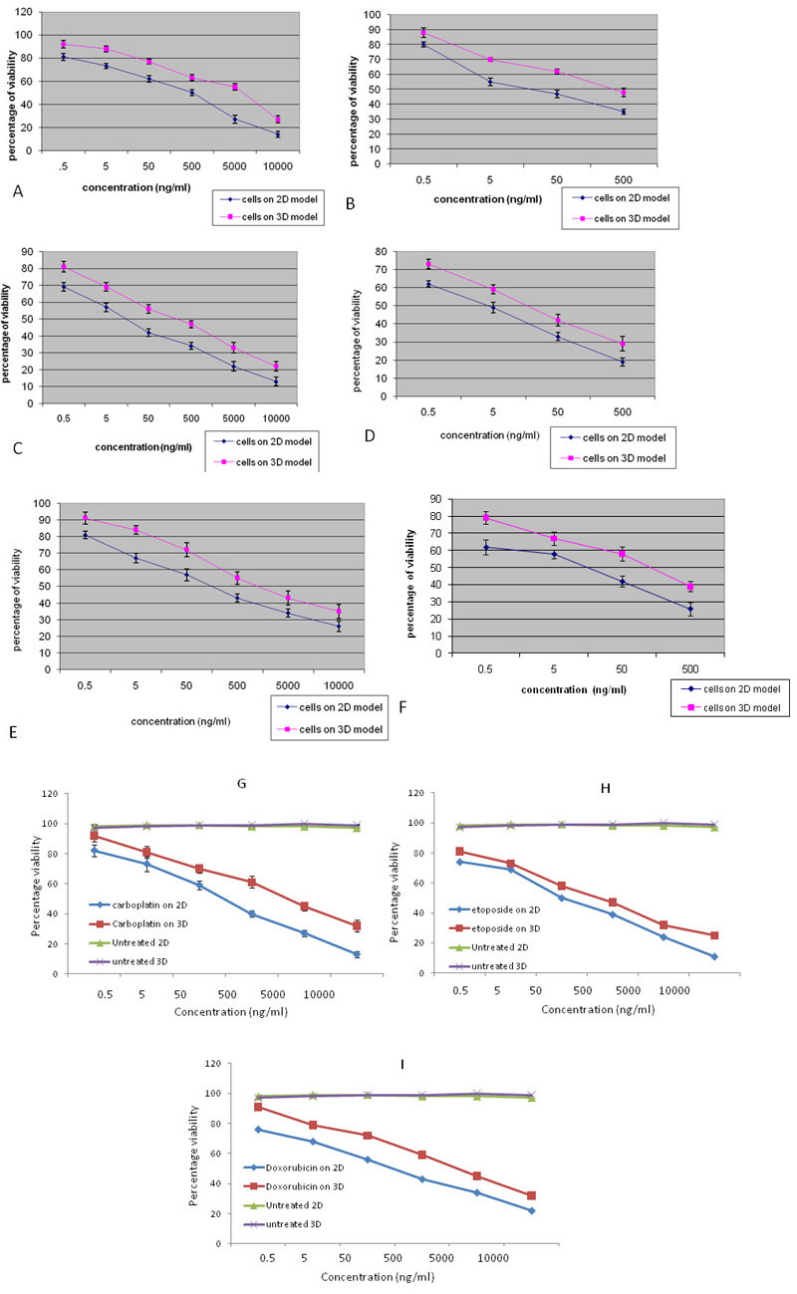
This figure shows the anti-proliferative effect of drug loaded nanoparticles on Y79 cells cultures in Two-dimensional (2-D) or three dimensional (3-D) patterns. **A**: Y79 cells co-cultured with microparticles (3-D) shows decreased sensitivity (p<0.001) to native carboplatin when compared to Y79 cells cultured without microparticles (2-D). **B**: Y79 cells co-cultured with microparticles (3-D) shows decreased sensitivity (p<0.0001) to Carboplatin loaded nanoparticles when compared to Y79 cells cultured without microparticles (2-D). **C**: Y79 cells co-cultured with microparticles (3-D) shows decreased sensitivity (p<0.001) to native etoposide when compared to Y79 cells cultured without microparticles (2-D). **D**: Y79 cells co-cultured with microparticles (3-D) shows decreased sensitivity (p<0.001) to etoposide-loaded Np when compared to Y79 cells cultured without microparticles (2-D). **E**: Y79 cells co-cultured with microparticles (3-D) shows decreased sensitivity (p<0.001) to native doxorubicin when compared to Y79 cells cultured without microparticles (2-D). **F**: Y79 cells co-cultured with microparticles (3-D) shows decreased sensitivity (p<0.001) to doxorubicin loaded nanoparticles when compared to Y79 cells cultured without microparticles (2-D). **G**: Fresh retinoblastoma tumor cells co-cultured with microparticles (3-D) shows decreased sensitivity (p<0.001) to native carboplatin when compared to Y79 cells cultured without microparticles (2-D). **H**: Fresh retinoblastoma tumor cells co-cultured with microparticles (3-D) shows decreased sensitivity (p<0.0001) to native etoposide when compared to Y79 cells cultured without microparticles (2-D). **I**: Fresh retinoblastoma tumor cells co-cultured with microparticles (3-D) shows decreased sensitivity (p<0.001) to native doxorubicin when compared to Y79 cells cultured without microparticles (2-D). Error bars represent standard deviation obtained from triplicates.

Cytotoxicity of model anticancer drugs was determined in Y79/RB cells grown with or without PLGA–gelatin microparticles following 48 h of drug treatment. Cell numbers were similar in both models at the time of treatment, and the inhibition in cell growth was calculated according to the respective untreated controls. IC_50_ values of drugs and drug-loaded nanoparticles were significantly higher in Y79 cells with PLGA–gelatin microparticles than in cells grown without PLGA–gelatin microparticles (Figure 7). The differences in the IC_50_ values observed were 4.5 to 21.8 fold depending upon the drug and drug-loaded nanoparticles (Table 2). Similarly, we observed significantly higher IC_50_ values of native drugs in RB tumor cells with PLGA–gelatin microparticles compared to RB cells grown without PLGA–gelatin microparticles (Figure 7G-I). The differences in the IC_50_ values of native drugs in RB tumor cells observed were 2.4 to 13.4 fold (Table 2).

**Table 2:** Comparison of IC_50_ values of respective native drugs and drug loaded nanoparticles between Y79 cells co-cultured with microparticles (3-D0 and Y79 cells cultured without microparticles (2-D).

	**IC_50_**		
**Drug**	**2-D**	**3-D**	**Fold change**
Native doxorubicin	154.8	1202	7.76
Np doxorubicin	9.54	102.3	10.7
Native etoposide	17.66	159.2	9.01
Np etoposide	3.86	17.37	4.5
Native carboplatin	138	1584	11.47
Np carboplatin	28.84	630	21.84

### Drug uptake by cells with or without poly(D,L)-lactide-co-glycolide-gelatin microparticles

**Figure 8:**
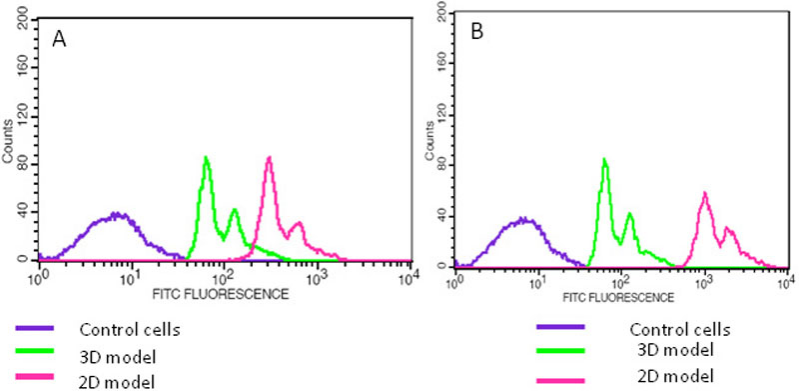
This figure shows the uptake of doxorubicin drug by Y79 cells either or not co-cultured with microparticles using flow cytometry. **A**: The flow cytometry histogram shows decreased uptake of drug by Y79 cells co-cultured with microparticles (3-D) when compared to Y79 cells cultured without microparticles at 4 h. **B**: Flow cytometry histogram shows decreased uptake of a drug by Y79 cells co-cultured with microparticles (3-D) when compared to Y79 cells cultured without microparticles at 24 h. The x-axis represent fluorescein isothiocyanate (FITC) intensity and the y-axis represents cell counts.

After treatment with doxorubicin (2,500 ng/ml), significantly fewer (p<0.05) cells with PLGA–gelatin microparticles exhibited drug uptake compared to cells grown without microparticles. The difference in fluorescence intensity was 4.4 fold at 4 h (Figure 8A), which increased to 15.9 fold at 24 h (Figure 8B).

### Microarray analysis

**Figure 9:**
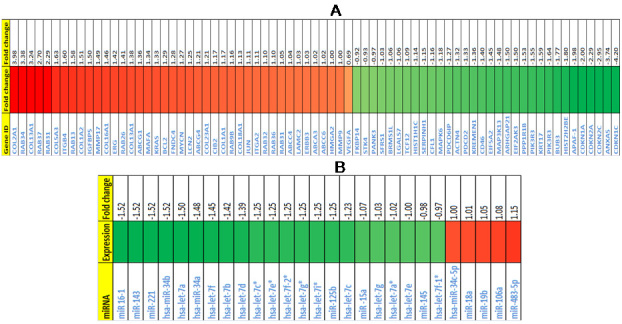
This figure represents microarray heat map displaying deregulated genes and microRNAs in Y79 cells either or not co-cultured with microparticles. **A**: Heat map shows significantly upregulated and down-regulated genes in Y79 cells co-cultured with microparticles (3-D) when compared with Y79 cells cultured without microparticles (2-D). **B**: Heat map shows significantly upregulated and down-regulated microRNAs in Y79 cells co-cultured with microparticles (3-D) when compared with Y79 cells cultured without microparticles (2-D). Green color represents upregulated genes or microRNAs and red color represents down-regulated genes or microRNAs. Heat map shows gene IDs or microRNA names and their respective expression fold changes in Y79 cells co-cultured with microparticles (3-D) when compared with Y79 cells cultured with microparticles (2-D).

Results of the cDNA microarray revealed changes in the expression of 4,490 genes (overexpression of 2,490 genes; downregulation of 2,000 genes) in cells grown with PLGA–gelatin microparticles compared to those grown without microparticles (Figure 9A). Only genes with p≤0.05 and a log ratio of at least 2.0 for upregulation (≥1 fold) and a log ratio of 0.5 for downregulation (≤0.5 fold), keeping a median log ratio of 1 in both biologic replicates, were considered for expression analysis [11]. The important upregulated (Table 3 and Table 4) and downregulated (Table 5) genes are provided. We classified the altered transcripts based on their biologic roles. The data also identified several candidate genes that are found to be highly overexpressed in retinoblastoma tumors and that keep tumors in a proliferation state, such as *Homo sapiens* v-myc myelocytomatosis viral-related oncogene (MYCN) [12,13], *H. sapiens* jun oncogene (Jun) [11], *H. sapiens* v-erb-b2 (ERBB3) [14], and *H. sapiens* insulin-like growth factor binding protein 5 (IGFBP5) [15]. We also found enhanced expression of collagen, integrins, fibronectin, and laminin, which crosslink and stiffen the tissue stroma to promote transformation, tumor growth, motility, and invasion and enhance cancer cell survival [16].

**Table 3:** Showing the upregulated genes in Y79 cells grown with microparticles compared to cells without microparticles.

**Gene ID**	**Gene name**	**Chromosome location**	**Fold change**	**significant p-value <0.05**	**Category**
*COL2A1*	Homo sapiens collagen, type II, alpha 1 [NM_001844]	chr12	3.9837	0.0007	Extracellular matrix protein
*COL1A1*	H.sapiens mRNA for prepro-alpha1(I) collagen. [Z74615]	17q21.33	1.1663	0.0026	Extracellular matrix protein
*COL1A2*	Homo sapiens collagen, type I, alpha 2 (COL1A2), mRNA [NM_000089]	7q21.3	1.5104	0.0496	Extracellular matrix protein
*COL13A1*	Homo sapiens collagen, type XIII, alpha 1 [NM_005203]	chr10	3.2391	0.0337	Extracellular matrix protein
*COL5A3*	Homo sapiens collagen, type V, alpha 3 [NM_015719]	chr19	1.6284	0.0145	Extracellular matrix protein
*COL16A1*	Homo sapiens collagen, type XVI, alpha 1 [NM_001856]	chr1	1.4568	0.0005	Extracellular matrix protein
*COL13A1*	Homo sapiens collagen, type XIII, alpha 1 [NM_005203]	chr10	1.3769	0.0337	Extracellular matrix protein
*COL23A1*	Homo sapiens collagen, type XXIII, alpha 1 [NM_173465]	chr5	1.206	0.0033	Extracellular matrix protein
*COL18A1*	Homo sapiens collagen, type XVIII, alpha 1 [NM_030582]	chr21	1.1288	0.0058	Extracellular matrix protein
*ITGB4*	Homo sapiens integrin, beta 4 [NM_000213]	chr17	1.6027	0.0008	Extracellular matrix protein
*CIB2*	Homo sapiens calcium and integrin binding family member 2 [NM_006383]	chr15	1.1749	0.0001	Extracellular matrix protein
*ITGA2*	Homo sapiens integrin, alpha 2 [NM_002203]	chr5	1.1054	0.0036	Extracellular matrix protein
*FNDC4*	Homo sapiens fibronectin type III domain containing 4 [NM_022823]	chr2	1.2791	0.005	Extracellular matrix protein
*LAMC2*	Homo sapiens laminin, gamma 2 [NM_005562]	chr1	1.0315	0	Extracellular matrix protein
*MYCN*	Homo sapiens v-myc myelocytomatosis viral related oncogene, neuroblastoma derived (avian) [NM_005378]	chr2	1.271	0.0003	oncogene
*JUN*	Homo sapiens jun oncogene [NM_002228]	chr1	1.108	0.0002	oncogene
*BCL2*	Human B-cell leukemia/lymphoma 2 (bcl-2) proto-oncogene [M13995]	chr18	1.2855	0.0007	oncogene
*ERBB3*	Homo sapiens v-erb-b2 erythroblastic leukemia viral oncogene homolog 3 (avian) [NM_001982]	chr12	1.03	0.0148	oncogene
*MAFA*	Homo sapiens v-maf musculoaponeurotic fibrosarcoma oncogene homolog [NM_201589]	chr8	1.3354	0.0009	oncogene
*ERG*	Homo sapiens v-ets erythroblastosis virus E26 oncogene homolog (avian) [NM_004449]	chr21	1.421	0.0185	oncogene
*RAB34*	Homo sapiens RAB34, member RAS oncogene family [NM_031934]	chr17	3.3803	0.0001	oncogene
*RAB37*	Homo sapiens RAB37, member RAS oncogene family [NM_175738]	chr17	2.695	0.0017	oncogene
*RAB31*	Homo sapiens RAB31, member RAS oncogene family [NM_006868]	chr18	2.2873	0.0051	oncogene
*RAB13*	Homo sapiens RAB13, member RAS oncogene family [NM_002870]	chr1	1.5812	0.0182	oncogene
*RAB26*	Homo sapiens RAB26, member RAS oncogene family [NM_014353]	chr16	1.4142	0.0004	oncogene
*LCN2*	Homo sapiens lipocalin 2 (oncogene 24p3) [NM_005564]	chr9	1.2526	0.0029	oncogene
*KRAS*	Homo sapiens v-K_i_-ras2 Kirsten rat sarcoma viral oncogene homolog, mRNA (cDNA clone IMAGE:5301134). [BC029545]	chr12	1.3272	0.0059	oncogene
*RAB9B*	Homo sapiens RAB9B, member RAS oncogene family, [BC018033]	chrX	1.1607	0.0197	oncogene
*RAB32*	Homo sapiens RAB32, member RAS oncogene family [NM_006834]	chr6	1.102	0.0124	oncogene
*RAB36*	Homo sapiens RAB36, member RAS oncogene family [NM_004914]	chr22	1.101	0.0043	oncogene
*RAB31*	Homo sapiens RAB31, member RAS oncogene family [NM_006868]	chr18	1.0456	0.0051	oncogene
*ABCG1*	Homo sapiens ATP-binding cassette, sub-family G (WHITE), [NM_207630]	chr21	1.3562	0.0018	ATP binding cassette
*ABCG4*	Homo sapiens ATP-binding cassette, sub-family G (WHITE), [NM_022169]	chr11	1.2119	0.0006	ATP binding cassette
*ABCC4*	Homo sapiens ATP-binding cassette, sub-family C (CFTR/MRP), member 4 [NM_005845]	chr13	1.0357	0.0104	ATP binding cassette
*ABCA3*	Homo sapiens ATP-binding cassette, sub-family A (ABC1), [NM_001089]	chr16	1.0245	0.0012	ATP binding cassette
*ABCC6*	Homo sapiens ATP-binding cassette, sub-family C (CFTR/MRP), [NM_001079528]	chr16	1.0239	0.0033	ATP binding cassette
*IGFBP5*	Homo sapiens insulin-like growth factor binding protein 5 (IGFBP5), mRNA [NM_000599]	chr2	1.5048	0.0001	Cell proliferation
*MMP17*	Homo sapiens matrix metallopeptidase 17 (membrane-inserted) (MMP17), mRNA [NM_016155]	chr12	1.4897	0.0036	invasion
*MMP 13*					

**Table 4:** Genes upregulated by more than 0.5 fold but less than 1 fold in Y79 cells grown with microparticles compared with cells grown without microparticles. These genes were considered based on the biologic and statistical significance (p<0.05).

**Gene ID**	**Gene name**	**Chromosome location**	**Fold change**	**significant p-value <0.05**	**category**
*MCC*	Homo sapiens mutated in colorectal cancers (MCC), mRNA [NM_002387]	chr5	0.5848	0.0041	oncogene
*HMGA2*	Homo sapiens high mobility group AT-hook 2 (HMGA2), transcript variant 1, mRNA [NM_003483]	chr12	0.8396	0.0035	oncogene
*MMP9*	Homo sapiens matrix metallopeptidase 9 (gelatinase B, 92 kDa gelatinase, 92 kDa type IV collagenase) (MMP9), mRNA [NM_004994]	chr20	0.6921	0.0041	Matrix metallo protease
*DDX1*	Homo sapiens DEAD (Asp-Glu-Ala-Asp) box polypeptide 1 (DDX1), mRNA [NM_004939]	chr2	0.6986	0.0003	oncogene

**Table 5:** Genes down-regulated in Y79 cell grown with microparticles (3-D) compared to those without microparticles (2-D).

**Gene name**	**Protein name**	**Fold change**	**P value**	**function**
*APAF-1*	Homo sapiens apoptotic peptidase activating factor 1 (APAF1), transcript variant 3, mRNA [NM_181861]	−1.9839	0.0012	Apaf-1, a cell-death effector that acts with cytochrome c and caspase-9 to mediate p53-dependent apoptosis
*PIK3R3*	Homo sapiens phosphoinositide-3-kinase, regulatory subunit 3 (p55, gamma) (PIK3R3), mRNA [NM_003629]	−1.644	0.0008	Pro-apoptosis.
*SFRS1*	Homo sapiens splicing factor, arginine/serine-rich 1 (splicing factor 2, alternate splicing factor) (SFRS1), transcript variant 1, mRNA [NM_006924]	−1.0263	0.0001	Pro-cell death
*FKBP14*	Homo sapiens FK506 binding protein 14, 22 kDa (FKBP14), mRNA [NM_017946]	−0.9243	0.0099	Pro-apoptosis
*PPP1R1B*	Homo sapiens protein phosphatase 1, regulatory (inhibitor) subunit 1B (dopamine and cAMP regulated phosphoprotein, DARPP-32) (PPP1R1B), transcript variant 1, mRNA [NM_032192]	−1.5266	0.0007	Pro-apoptosis
*ANXA5*	Homo sapiens annexin A5 (ANXA5), mRNA [NM_001154]	−3.7394	0.0002	Pro- apoptosis
*TCF12*	Homo sapiens transcription factor 12 (HTF4, helix–loop–helix transcription factors 4) (TCF12), transcript variant 4, mRNA [NM_207038]	−1.0896	0.0028	Pro apoptosis
*PDCD2*	Homo sapiens programmed cell death 2 (PDCD2), transcript variant 1, mRNA [NM_002598]	−1.3256	0.0011	Pro apoptosis
*ACTN4*	Homo sapiens actinin, alpha 4 (ACTN4), mRNA [NM_004924]	−1.3227	0	Pro apoptis
*MAPK6*	Homo sapiens mitogen-activated protein kinase 6 (MAPK6), mRNA [NM_002748]	−1.1824	0.0236	Pro apoptosis
*EIF5A2*	Homo sapiens eukaryotic translation initiation factor 5A2 (EIF5A2), mRNA [NM_020390]	−1.4533	0.0001	Pro apoptosis
*MAP3K13*	Homo sapiens mitogen-activated protein kinase kinase kinase 13 (MAP3K13), mRNA [NM_004721]	−1.4791	0.0138	Pro apoptosis
*HIST2H2BE*	Homo sapiens histone cluster 2, H2be (HIST2H2BE), mRNA	−1.8046	0.001	Pro apoptosis
*HIST1H1C*	Homo sapiens histone cluster 1, H1c (HIST1H1C), mRNA [NM_005319]	−1.1425	0.0002	Pro apoptosis
*PDCD6IP*	Homo sapiens programmed cell death 6 interacting protein (PDCD6IP), mRNA [NM_013374]	−1.2727	0.0073	Pro apoptosis
*CFL1*	Homo sapiens cofilin 1 (non-muscle) (CFL1), mRNA [NM_005507]	−1.1578	0.0068	Pro apoptosis
*EIF2AK3*	Homo sapiens eukaryotic translation initiation factor 2-alpha kinase 3 (EIF2AK3), mRNA [NM_004836]	−1.502	0.0013	Proapoptosis
*BUB3*	Homo sapiens mRNA for BUB3 budding uninhibited by benzimidazoles 3 isoform a variant, clone: hg01710. [AK226060]	−1.7701	0.003	Pro apoptosis
*ARHGAP21*	Homo sapiens Rho GTPase activating protein 21 (ARHGAP21), mRNA [NM_020824]	−1.4986	0.0001	Anti metastasis
*KRT17*	Homo sapiens keratin 17 (KRT17), mRNA [NM_000422]	−1.5924	0.0057	Anti metastasis
*CD46*	Homo sapiens CD46 molecule, complement regulatory protein (CD46), transcript variant a, mRNA [NM_002389]	−1.3955	0.0004	Anti metastasis
*SERPINH1*	Homo sapiens serpin peptidase inhibitor, clade H (heat shock protein 47), member 1, (collagen binding protein 1) (SERPINH1), mRNA [NM_001235]	−1.1452	0	Anti-metastasis
*BRMS1L*	Homo sapiens breast cancer metastasis-suppressor 1-like (BRMS1L), mRNA [NM_032352]	−1.0558	0	Anti-metastasis
*PANK3*	Homo sapiens pantothenate kinase 3 (PANK3), mRNA [NM_024594]	−0.9712	0.0019	Anti-metastasis
*LGALS7*	Homo sapiens lectin, galactoside-binding, soluble, 7 (galectin 7) (LGALS7), mRNA [NM_002307]	−1.0622	0.0002	Anti-metastasis
*STK4*	Homo sapiens serine/threonine kinase 4, mRNA (cDNA clone IMAGE:3950315), complete cds. [BC005231]	−0.9328	0.0002	Tumor supression
*PARP9*	Homo sapiens poly (ADP-ribose) polymerase family, member 9 (PARP9), mRNA [NM_031458]	−1.4829	0.038	Tumor supression
*CDKN1A*	Homo sapiens cyclin-dependent kinase inhibitor 1A (p21, Cip1) (CDKN1A), transcript variant	−2.0045	0.0027	Tumor supression
*CDKN1C*		−4.2038	0.0002	Tumor supression
*CDKN2C*	Homo sapiens cyclin-dependent kinase inhibitor 2C (p18, inhibits CDK4) (CDKN2C), transcript variant 1, mRNA [NM_001262]	−2.9462	0	Tumor supression
*CDKN2A*	Homo sapiens cyclin-dependent kinase inhibitor 2A (melanoma, p16, inhibits CDK4) (CDKN2A), transcript variant 3, mRNA [NM_058197]	−2.2861	0.0002	Tumor supression
*KREMEN1*	Homo sapiens kringle containing transmembrane protein 1 (KREMEN1), transcript variant 1, mRNA [NM_153379]	−1.3621	0.0034	Tumor supression
*SASH1*	Homo sapiens SAM and SH3 domain containing 1 (SASH1), mRNA [NM_015278]	−4.8233	0	Tumor supression
*RRM2*	Homo sapiens ribonucleotide reductase M2 polypeptide (RRM2), mRNA [NM_001034]	−1.309	0.0032	angiogenesis

### MicroRNA analysis

Results of the microRNA analysis revealed changes in the expression of 52 miRNAs (overexpression of 6 miRNAs; downregulation of 46 miRNAs) in cells grown with PLGA–gelatin microparticles compared to those grown without microparticles (Figure 9B). The important differentially regulated miRNAs are provided in Table 6. We classified the altered miRNAs based on their biologic roles (oncomirs and tumor suppressors). Our data has identified downregulated tumor suppressor miRNAs in cells with PLGA–gelatin microparticles, such as *let-7* miRNAs (let-7 family) and *miR-34a* (which may enhance retinoblastoma tumor proliferation) [17,18] and *miR-15a* and *miR-16*, which have been implicated in cell-cycle control and apoptosis [19]. We also identified upregulated miRNAs, such as miR-198, which may play significant roles in regulating retinoblastoma tumor genesis [20]; miR-18a and miR-19b, which are upregulated in various carcinomas and increased expression of this cluster contributes to cancer formation [21]; and miR-106a, which is a potential biomarker in the diagnosis of gastric carcinoma [22].

**Table 6:** MicroRNAs deregulated in Y79 cells grown with microparticles (3-D compared to cells without microparticles (2-D).

**miRNA**	**Fold change**	**Function**	**p-value**
miR-15a	−1.0695	TS	0.0294
miR-16–1	−1.5218	TS	0.0429
miR-143	−1.5218	TS	0.0429
miR-145	−0.9849	TS	0.0168
hsa-let-7a	−1.5014	TS	0.0231
hsa-let-7a*	−1.0175	TS	0.0010
hsa-let-7b	−1.4151	TS	0.0134
hsa-let-7c	−1.2288	TS	0.0346
hsa-let-7c*	−1.2534	TS	0.0429
hsa-let-7d	−1.3859	TS	0.0132
hsa-let-7e	−1.0035	TS	0.0480
hsa-let-7e*	−1.2534	TS	0.0429
hsa-let-7f	−1.4460	TS	0.0229
hsa-let-7f-1*	−0.9678	TS	0.0054
hsa-let-7f-2*	−1.2534	TS	0.0429
hsa-let-7g	−1.0258	TS	0.0152
hsa-let-7g*	−1.2534	TS	0.0429
hsa-let-7i*	−1.2534	TS	0.0429
hsa-miR-125b	−1.2534	TS	0.0429
hsa-miR-221	−1.5218	TS	0.0429
hsa-miR-19b	1.0548	OG	0.0386
hsa-miR-106a	1.0828	OG	0.0011
hsa-miR-18a	1.0100	OG	0.0053
hsa-miR-34a	−1.4787	TS	0.0159
hsa-miR-34b	−1.5218	TS	0.0429
hsa-miR-34c-5p	1.0000	OG	0.0483
hsa-miR-483–5p	1.1500	OG	0.0317

TS: tumor suppressor, OG: oncogene.

### Real-time quantitative RT–PCR

**Figure 10:**
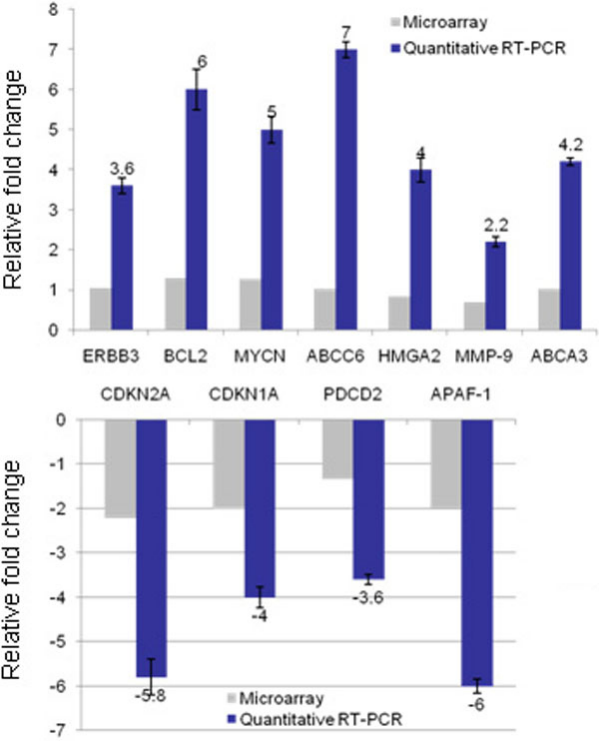
This figure shows the real time quantitative PCR validation of selected genes from microarray results. **A**: Graph shows real time quantitative PCR analysis showing upregulated genes (dark bars) in Y79 cells co-cultured with microparticles (3-D) when compared with Y79 cells cultured without microparticles (2-D). The fold change of respective genes in microarray was also displayed (gray bars) for comparison. The genes that were upregulated in microarray analysis were also found to be upregulated in quantitative real time PCR analysis. **B**: Graph shows real time quantitative PCR analysis showing down-regulated genes (dark bars) in Y79 cells co-cultured with microparticles (3-D) when compared with Y79 cells cultured without microparticles (2-D). The fold change of respective genes in microarray was also displayed (gray bars) for comparison. The genes that were down-regulated in microarray analysis were also found to be down-regulated in quantitative real time PCR analysis. The error bars represent the data in triplicates.

Seven upregulated genes-erythroblastic leukemia viral oncogene homolog 3(*ERBB-3*), B-cell leukemia/lymphoma 2 (*BCL-2*), v-myc myelocytomatosis viral related oncogene, neuroblastoma derived (*MYCN*), ATP-binding cassette, sub-family C-member 6 (*ABCC6),* High mobility group AT-hook 2 (*HMGA-2*), Matrix metallopeptidase 9 (*MMP-9*), ATP-binding cassette, sub-family A-member 3 (*ABCA-3*) and four down-regulated genes- Cyclin-dependent kinase inhibitor 2A (*CDKN-2*), Cyclin-dependent kinase inhibitor 1A (*CDKN-1A*), Programmed cell death 2 (*PDCD-2*) and apoptotic peptidase activating factor 1 (*APAF-1*)-from the microarray data have been confirmed by real-time quantitative RT–PCR. The results are consistent with the microarray data. The fold changes of all the genes studied was only slightly higher when measured using quantitative RT–PCR compared to the microarray fold changes, reflecting the better dynamic range of quantitative RT–PCR (Figure 10).

## Discussion

Owing to excellent biocompatibility and high mechanical strength, the biodegradable polyester PLGA has been frequently used (as biomaterial) for drug delivery systems as well as for tissue engineering applications. However, due to its hydrophobic properties it is difficult to wet the microparticles (constituent of PLGA) in cell culture medium and hence this limits the adhesion of proliferating cells [7,23]. To circumvent this problem, our formulated microparticles were coated with a hydrophilic polymer of natural origin, i.e., gelatin. Further, with a view to promoting better cell adhesion, we incorporated PVA in the internal phase of the primary emulsion. Here, the amphiphilic PVA could anchor at the interface of the microparticle with its hydrophobic portion embedded in the PLGA matrix structure and its hydrophilic portion available for hydration in the cell culture media [7,24]. Furthermore, the anchored PVA can provide functional OH groups, which have been reported as being helpful in cell attachment [25]. Beside PVA, another polymer—chitosan—was incorporated in the internal matrix structure for better cell growth of cultured cells in our formulated microparticles. Here, chitosan can support cell attachment owing to its chemical structure resembling glycosaminoglycans, which is abundant in extracellular matrix components [26]. Similarly, sucrose was added (in the internal phase of the primary emulsion sucrose) to increase the surface tension of water [27]. Formation of larger water droplets upon leaching out can generate more porous microparticles, which in turn provided more surface area to promote cell adhesion, proliferation, and growth, as found in our study.

Tumor progression ensues within a 3-D microenvironment, and the metastatic potential of tumor cells is believed to be regulated by interactions between the tumor cells and their extracellular environment (i.e., ECM). These interactions can be modified by the accumulation of genetic changes and by the transient alterations in gene expression induced by the local tumor microenvironment, which consists of cellular and noncellular components. Noncellular aspects of the tumor microenvironment, such as ECM, have been shown to influence tumor progression either directly by destabilizing tissue integrity and promoting tumor cell motility, invasion, and survival [28] or indirectly by inducing tumor angiogenesis and enhancing tumor cell survival and selection [29,30]. 3-D tumor models using different substrates, such as Matrigel [31], laminin-rich extracellular matrix [1], irradiated HeLa cells [32], or teflon membrane [33,34], have been developed to study the various aspects of, for example, tumor biology, phenotypic alterations, and invasive and migratory behavior of cells. Y79 cells when grown on porous microparticles tend to synthesize extracellular matrix proteins, such as collagens (including type I and III) and fibronectin, which together contribute to the mechanical strength of the tissue. Collagenase assay analysis also showed significantly higher collagen synthesis by Y79 cells grown on microparticles. It is known that in tumor masses not all cancer cells are exposed to the same concentration of drugs because of poor drug diffusion through the extracellular matrix of the tumor [35]; this causes the tumor to relapse or develop drug resistance [8]. Our study also demonstrated significantly slow diffusion and heterogeneous distribution of drugs and drugs encapsulated in nanoparticles in the cells grown on PLGA–gelatin microparticles compared to cells grown without. The important application of our study will be to predict the efficacy of drugs in vivo. Our study also reports significant genomic variations in the cells when grown with porous microparticles. Enhanced expression of the collagen family members laminin and fibronectin were observed in our study, which strongly supports our initial findings. In response to collagen synthesis, we observed increased expression of matrix metalloproteinases (MMPs), such as *MMP-13* [36] and *MMP-9* [37,38], the latter facilitates the tumor cells to invade the extracellular matrix. Several candidate genes, such as *MYCN*, *ERBB3*, *JUN*, and *IGFBP5*, were also significantly upregulated, which keeps the tumor in a proliferative state [11-15].

Changes in the upregulation of genes were coupled with downregulation of a few genes that function as tumor suppressor/apoptotic inducers. *Homo sapiens APAF-1*, transcript variant 3, mRNA (*Apaf-1*) is a central component of the intrinsic pathway of apoptosis and is important for cellular responses to DNA damage [39]. When DNA is damaged by chemotherapeutic agents, oncogenic stimuli, ultraviolet and ionizing radiation, and hypoxia, cytochrome *c* is released from the mitochondria, binds to Apaf-1 in the cytosol, and in association with dATP/ATP facilitates a conformational change of Apaf-1 to expose its CARD domain [40]. It has been shown that inactivation of Apaf-1 substantially reduces the number of cells required to form tumors in nude mice transplanted with Myc/Ras-transformed fibroblasts, implying that Apaf-1 actually acts as a tumor suppressor [41]. *Homo sapiens* cyclin-dependent kinase inhibitor 2A (CDKN2A; melanoma, p16, inhibits CDK4), a major CDK inhibitor, is the product of a tumor-suppressor gene that has been found inactivated in different cancer types. CDK inhibitor p21 (CDKN1A) is induced by both p53-dependent and p53-independent mechanisms following stress, and induction of p21 may cause cell-cycle arrest; as a proliferation inhibitor, p21 is poised to play an important role in preventing tumor development [42]. This notion is supported by data indicating that p21 null mice are more prone to spontaneous and induced tumorigenesis and that p21 synergizes with other tumor suppressors to protect against tumor progression in mice. *Homo sapiens* programmed cell death 2 (PDCD2), transcript variant 1, mRNA promotes apoptosis in several human lymphomas. Repression of PDCD2 expression by BCL6, which in turn leads to downregulation of apoptosis, is one mechanism involved in BCL6-associated lymphomatous transformation [43].

MicroRNAs are short (20–23 nucleotides in length) noncoding RNAs that mediate coordinated cellular programs by posttranscriptional mRNA silencing and protein translation inhibition [44]. A growing number of miRNAs have been implicated in promoting or suppressing tumorigenesis in a variety of tissues [45,46], suggesting that they may play a role as a novel class of oncogenes or tumor suppressor genes. Our study revealed alterations in miRNA expression when a Y79 cell line is grown with microparticles.

We observed enhanced expression of oncogenic microRNAs, such as miR-18a, miR-19b [47], miR-106a [22], and miR-198 [20], which subsequently promote proliferation, inhibit apoptosis, and promote tumor angiogenesis. There was also decreased expression in miRNAs, such as let-7 family [48], miR 15a and miR16–1 [49], and miR 34a [50] which act as tumor suppressors in various carcinomas, including retinoblastoma. Let-7 downregulates oncogenes, such as Ras, Myc, and HMGA2, and inhibits proliferation and tumorigenesis when ectopically expressed [51].

In this study, we fabricated porous microparticles with a composite interface containing PLGA and chitosan, which causes cancer cells to develop into a tumor-like structure in vitro. The drug efficacy was significantly lower in cells grown with microparticles than in cells grown without microparticles, suggesting a role of cellular architecture on drug uptake, distribution, and efficacy. Our tumor model could potentially be applicable in developing effective drugs based on a better understanding of the role of chemical, biologic, and physical parameters in the process of drug diffusion through the tumor mass, drug retention, and therapeutic outcome. A correlation between the drug effects seen in the tumor model to the in vivo efficacy would further establish the usefulness of our model in drug discovery. In conclusion, we developed a novel, retinoblastoma, in vitro, 3-D model that could be used for evaluating chemotherapeutic drugs in the future.
